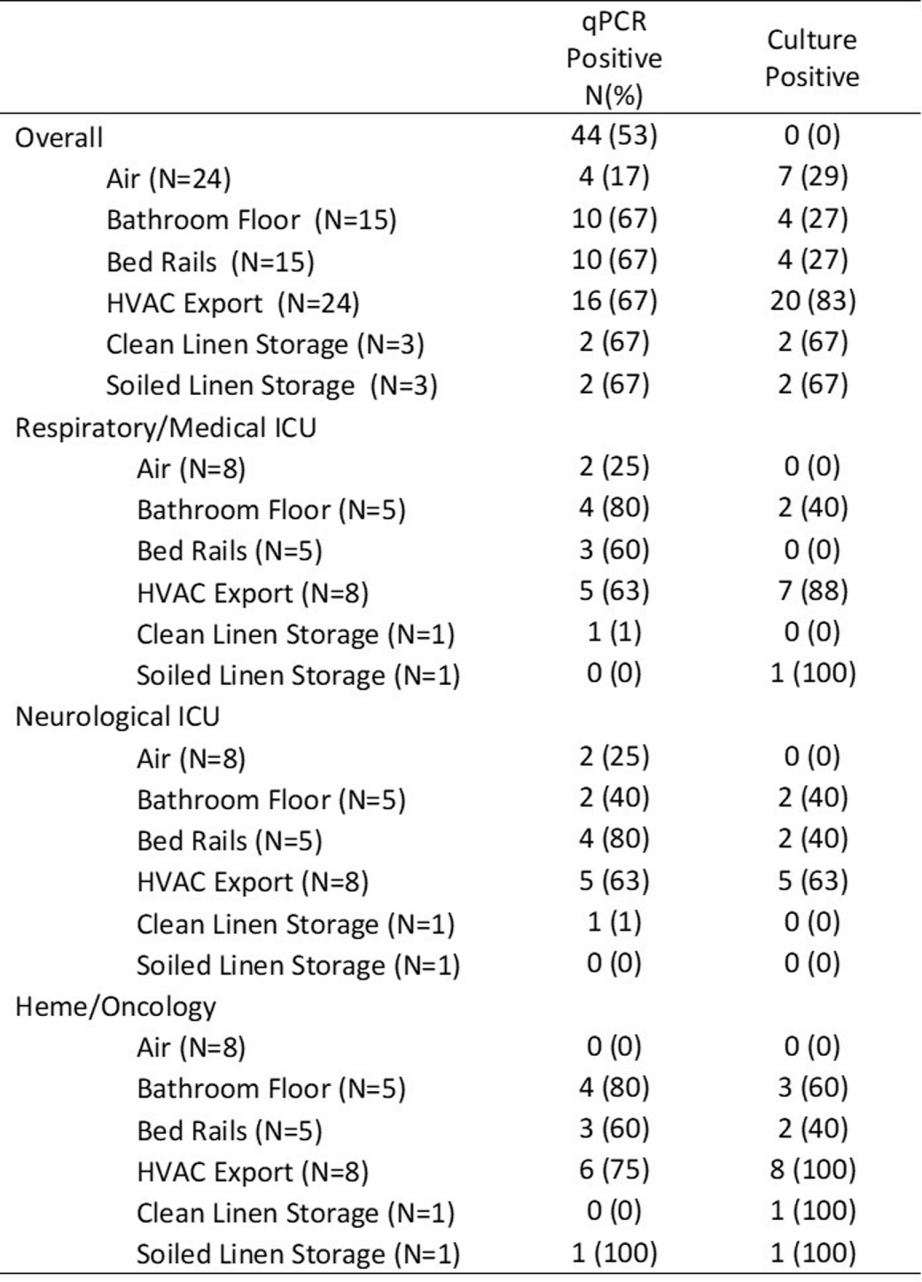# Environmental Fungal Contamination Characterization of Three Inpatient Units Utilizing Optimized Detection Methods

**DOI:** 10.1017/ash.2024.243

**Published:** 2024-09-16

**Authors:** Bobby Warren, Guerbine Fils-aime, Amanda Graves, Aaron Barrett, Matthew Steigel, Becky Smith, Ilan Schwartz, Deverick Anderson

**Affiliations:** Duke Center for Antimicrobial Stewardship and Infection Prevention; Duke University Hospital; Duke University Medical Center

## Abstract

**Background:** Environmental sampling and detection methods for fungi in healthcare settings are not well-established. We previously refined methods for fungal sampling and detection in a controlled laboratory environment and aimed to validate them in a real-world healthcare setting. **Methods:** We performed a microbiological analysis of air and surfaces in three inpatient units at a tertiary care center. Surface samples were obtained with foam sponges from 3 locations in patient rooms (Patient bedrails, bathroom floor, HVAC export) and 5 locations in units (HVAC exports 3x, clean linen storage, soiled linen storage). Air samples were taken with an active air sampler directly below HVAC exports. Sponges were processed using the stomacher technique. Samples underwent DNA extraction followed by qPCR with FungiQuant primers targeting the 18S rRNA gene. Amplicons from positive samples were sequenced (NextSeq 1000, 300bp PE) and SmartGene databases were used to interpret sequence data. For comparison to culture methods, samples were also plated onto Sabouraud and HardyCHROM Candida + auris medias. Fungal growth underwent DNA extraction, 18S PCR and Sanger sequencing for genus and species identification. **Results:** A total of 85 samples were obtained, from 15 patient rooms and three units resulting in 61 surface and 24 air samples. Patients in study rooms had a median age of 53, 9 (60%) were male, and no patients had an invasive fungal infection during their hospital encounter. 44 (53%) and 39 (46%) samples were positive for fungi via qPCR and culture, respectively. Of the 44 positive qPCR samples, microbiome analyses identified at least one fungi to the species, genus and family levels in 43 (98%), 28 (64%), 18 (41%) samples, respectively (Table 1). 114 total isolates were identified of which the most common were Mallassezia restricta (30 [26%]), Malassezia globose (29 [25%]), and Pennicillium paradoxum (4 [4%]). 39 genera were identified of which the most common were Mucor (19 [49%]) and Candida (8 [21%]). Of the 39 culture positive samples, 90 total isolates were recovered. The most common species were Paradendryphiella arenariae (19 [21%]), Aspergillus niger (12 [13%]) and Penicillium commune (12 [13%]). **Conclusion:** These results demonstrate the presence of diverse fungal species in both air and surface samples across inpatient units. Higher sensitivity was noted utilizing qPCR, however, identified genera and species were markedly different between qPCR and culture methods. Larger studies are needed to assess the efficacy of qPCR for fungal detection in the healthcare environment.

**Figure:**